# GABA_A_ receptor subtypes and benzodiazepine use, misuse, and abuse

**DOI:** 10.3389/fpsyt.2022.1060949

**Published:** 2023-01-12

**Authors:** Elif Engin

**Affiliations:** ^1^Stress Neurobiology Laboratory, Division of Basic Neuroscience, McLean Hospital, Belmont, MA, United States; ^2^Department of Psychiatry, Harvard Medical School, Boston, MA, United States

**Keywords:** benzodiazepines (BDZs), drug abuse, GABA_A_ receptor, withdrawal, tolerance, reward, dependence

## Abstract

Benzodiazepines have been in use for over half a century. While they remain highly prescribed, their unfavorable side-effect profile and abuse liability motivated a search for alternatives. Most of these efforts focused on the development of benzodiazepine-like drugs that are selective for specific GABA_A_ receptor subtypes. While there is ample evidence that subtype-selective GABA_A_ receptor ligands have great potential for providing symptom relief without typical benzodiazepine side-effects, it is less clear whether subtype-selective targeting strategies can also reduce misuse and abuse potential. This review focuses on the three benzodiazepine properties that are relevant to the DSM-5-TR criteria for Sedative, Hypnotic, or Anxiolytic Use Disorder, namely, reinforcing properties of benzodiazepines, maladaptive behaviors related to benzodiazepine use, and benzodiazepine tolerance and dependence. We review existing evidence regarding the involvement of different GABA_A_ receptor subtypes in each of these areas. The reviewed studies suggest that α1-containing GABA_A_ receptors play an integral role in benzodiazepine-induced plasticity in reward-related brain areas and might be involved in the development of tolerance and dependence to benzodiazepines. However, a systematic comparison of the contributions of all benzodiazepine-sensitive GABA_A_ receptors to these processes, a mechanistic understanding of how the positive modulation of each receptor subtype might contribute to the brain mechanisms underlying each of these processes, and a definitive answer to the question of whether specific chronic modulation of any given subtype would result in some or all of the benzodiazepine effects are currently lacking from the literature. Moreover, how non-selective benzodiazepines might lead to the maladaptive behaviors listed in DSM and how different GABA_A_ receptor subtypes might be involved in the development of these behaviors remains unexplored. Considering the increasing burden of benzodiazepine abuse, the common practice of benzodiazepine misuse that leads to severe dependence, and the current efforts to generate side-effect free benzodiazepine alternatives, there is an urgent need for systematic, mechanistic research that provides a better understanding of the brain mechanisms of benzodiazepine misuse and abuse, including the involvement of specific GABA_A_ receptor subtypes in these processes, to establish an informed foundation for preclinical and clinical efforts.

## 1. Introduction

Benzodiazepines (BDZs) have been in use since 1960s and are still prescribed at high rates with over 90 million prescriptions dispensed in the US alone each year (1). In 2015, one in eight US adults reported BDZ use within the past year, further illuminating the widespread use of BDZs. Studies from other countries indicate comparable rates of prescribed or non-medical BDZ use despite some variation in rates and in the specific subpopulations (e.g., the elderly) where BDZ use is most common (2–8).

BDZs achieve their therapeutic effects through the allosteric modulation of gamma amino butyric acid type A receptors (GABAARs). GABAARs are postsynaptic pentameric complexes, with the subunits comprising the pentamere drawn from a subunit repertoire of at least 19 subunits (α1-6, β1-3, γ1-3, δ, ε, θ, π, ρ1-3). Most GABAARs in the brain are composed of 2 α, 2 β and one γ or δ subunit, with the specific subunit composition influencing receptor kinetics, subcellular localization, and anatomical distribution of the receptor in the brain, as well as its pharmacological properties with regards to its modulation by different drug classes (9–12). GABA binding to binding sites at the interface of α and β subunits leads to the opening of the chloride channel at the center of the pentamere, allowing chloride movement between the intracellular and extracellular spaces. In the adult brain, this usually results in chloride influx to the cell and hyperpolarization, while in the immature brain [and possibly in the mature brain under certain pathological conditions; (13)], the opening of the channel leads to chloride efflux and depolarization. BDZ binding sites are distinct from the GABA-binding site and are located at the interface of the α and γ subunits on GABAARs containing the α1, α2, α3, or α5 subunits (α1GABAAR, α2GABAAR, α3GABAAR, and α5GABAAR). Thus, BDZs bind a subset of GABAARs, at a site distinct from the GABA-binding site, and their effect is to increase the frequency of chloride channel opening at a given GABA concentration, causing a leftward shift in the GABA dose-response curve without altering the maximal response.

BDZs have anxiolytic, sedative, hypnotic, amnestic, anticonvulsant, myorelaxant effects (9). While this heterogenous effect profile has made it possible for BDZs to be used for a wide range of indications and in different settings, the desired effects in one setting are often viewed as undesired side-effects in another setting (e.g., sedation and anterograde amnesia are highly desirable effects when BDZs are used in a peri-surgical setting but are highly undesirable when they are used as anxiolytics in the treatment of generalized anxiety disorder).

Considering the apparent functional relevance of the subunit composition of GABAARs to receptor properties and anatomical location, it was postulated that the different behavioral effects of BDZs may be mediated by their positive modulation of different GABAAR subtypes. Findings from early studies indeed indicated that BDZ modulation of α1GABAARs is required for the sedative effects (14), while BDZ modulation of α2GABAARs is required for the anxiolytic-like effects of BDZs (15). Continued work in this area not only confirmed and further expanded the association of specific behavioral effects with specific GABAAR subtypes (16–25), but uncovered new, previously unappreciated indications for subtype-selective GABAAR modulation (26–30).

The above studies, many of which were carried out in genetically modified mice due to a lack of subtype-specific pharmacological agents, demonstrated the possibility of developing subtype-specific agents that would have efficacy for specific indications without the undesirable effects of BDZs. Efforts to develop subtype-selective GABAAR modulators have yielded a large number of drugs in the last 30 years [For recent reviews, see (31, 32)]. While no truly subtype-specific drug has been developed to date, several compounds with subtype-selective affinity or subtype-selective efficacy have been investigated in preclinical studies for their behavioral effects, with a few of them also making it to clinical trials. The below sections aim to answer the question of whether these subtype-selective compounds would have reduced abuse and dependence liability compared to classical BDZs by summarizing relevant findings from preclinical studies.

## 2. Benzodiazepine abuse and misuse

DSM-5-TR (33) criteria for Sedative, Hypnotic, or Anxiolytic Use Disorder (pp. 620–621) focus on a number of problematic drug-related behaviors many of which can also be studied in preclinical work. The criteria can be roughly categorized as those that indicate *loss of control over use* (i.e., using the drug in larger doses or for a longer time than intended, continuing use despite negative consequences, failed attempts to reduce or stop use), *expenditure of significant time and effort for drug related activities, often at the expense of other desirable activities* (e.g., time/effort/money spent in acquiring the drug, recovering from drug effects, giving up on other activities in favor of using the drug, not being able to focus on other activities due to craving, failure to fulfill major obligations at work, home, school, other social settings, due to drug use), *risky drug use* (e.g., recurrent use in physically hazardous situations such as driving under the influence, taking risks to acquire the drug), and *pharmacological criteria* (i.e., development of tolerance and withdrawal). Having only 2 of the 11 listed symptoms is sufficient for diagnosis, with the presence of 2–3 symptoms considered “mild”, 4–5 symptoms “moderate”, and 6 or more symptoms “severe”.

While the DSM criteria outline the typical *behavioral presentations* of BDZ abuse and misuse, research on medical and non-medical use of BDZs reveals the most common *reasons* underlying BDZ abuse and misuse.

While some recreational users of BDZs use BDZs alone for their alcohol-like euphoric effects, BDZs are more often abused in combination with other drugs, most commonly opioids, to supplement the high (34–36). These users typically use BDZs at higher doses than the common therapeutic range (37) and as suggested by the recent popularity of fast-acting designer BDZs in illicit drug markets, they may prefer faster and shorter acting BDZs (38). Another common use of BDZs among illicit polydrug users is to use the BDZs as a way of managing the anxiety and irritability commonly experienced as a part of the withdrawal from the primary drug when regular access is disrupted, or managing anxiety experienced due to co-occurring psychiatric conditions (34, 39).

Misuse of BDZs in medical settings involves the use of BDZs for different indications, at different doses, and/or for longer periods of time than recommended. Off-label prescription of BDZs, particularly for indications such as post-traumatic stress disorder, obsessive-compulsive and related disorders, and mood disorders is common (40–43). While this is a concern, it should be noted that off-label prescription of medications for different indications than those approved is common practice for many drugs and is not specific to BDZs.

The second concern with BDZ misuse is patients using BDZs at higher doses that recommended, particularly when used long-term. While well-documented development of tolerance to the effects of BDZs would support the expectation that patients would escalate dose with long-term use, there has been relatively little empirical evidence to support consistent escalation of BDZ dose, even among long-term BDZ users (44, 45). This may be due to the fact that tolerance develops primarily to the sedative effect of BDZs, which is often viewed as an undesirable side-effect by individuals who take BDZs for anxiety-related indications, while tolerance to the anxiolytic effect is either small and delayed or non-existent in humans (46).

While off-label use or dose escalation do not seem to be major concerns for BDZ misuse, extended use is a significant issue. Current recommended length of treatment with BDZs is 2–4 weeks, with no BDZ approved for use for more than 4 months. Yet, many patients are prescribed BDZs for months, years, decades, sometimes indefinitely (47–53). More alarmingly, while the number of new BDZ prescriptions remained stable between 2005 and 2015, there was a 50% increase in renewed prescriptions during the same period, suggesting a specific increase in this problematic, longer-term use (54). Aside from continued need for therapeutic relief, withdrawal symptoms are the primary reason for long-term BDZ use.

In 2020, FDA issued a requirement to update the Boxed Warning on BDZs, indicating that following chronic use of BDZs over several days or weeks, abrupt cessation or dose reduction of BDZs can cause severe withdrawal symptoms, including seizures (1). Indeed, studies indicate that withdrawal symptoms can continue for months, even years (55). In a recent Internet study, 60–85% of individuals reported having moderate to very severe symptoms in different life domains while tapering off BDZs, with 54% of them reporting suicidal thoughts (55). The challenges involved in discontinuing BDZs were present even when tapering was done in a clinical setting where the withdrawal symptoms were closely managed (56, 57).

In summary, two major reasons for BDZ abuse and misuse are the reward-related effects of BDZs, mainly related to abuse, and physical dependence, as defined by the presence of a withdrawal syndrome upon discontinuation, which is the primary underlying factor for misuse, with likely involvement in abuse as well. As noted earlier, the efforts to develop GABAAR subtype selective compounds have been motivated by the idea of developing GABAergic therapeutics without the unfavorable side-effect profile of classical BDZs. Thus, a highly significant question is whether GABAAR subtype-specific compounds, if developed, would have the same abuse and misuse liability as BDZs. To start answering this question, we review evidence regarding the involvement of specific GABAAR subtypes in behaviors relevant to the 3 main domains of DSM-5-TR criteria for Sedative, Hyponotic, and Anxiolytic Use disorder: Reward-related effects of BDZs which support persistent drug-seeking, development of maladaptive behaviors associated with BDZ use, and development of BDZ tolerance and withdrawal.

## 3. GABAAR subtypes and reward-related effects of BDZs

Based on the DSM criteria provided above, it is possible to inquire into the rewarding effects of BDZs at multiple levels. For any compound to be used by choice or abused, it should first serve as a reinforcer, that is, its administration should increase the likelihood of behaviors that preceded it and/or were causally linked to it. The simplest form of this would be a preference for BDZs over alternatives when the two come at equal and negligible cost. For instance, rodents drink more from the bottle containing the water-soluble BDZ midazolam, when midazolam and water are provided in a two-bottle choice setup in their home-cages (24, 58–60). A related concept is drug-seeking behavior: BDZs support associative learning in a conditioned place preference paradigm where animals spend more time in the BDZ-associated chamber of a two-chamber apparatus during the drug-free test session (61). The second level would be the question of willingness to expend effort to acquire the drug. BDZs are self-administered in tests where animals have to engage in operant behaviors (e.g., press a lever) to receive the drug (34, 62) and increase the level of effort the animals are willing to expend to receive a brain stimulation reward in intracranial self-stimulation (ICSS) studies [i.e., reward enhancement; (24, 63, 64)]. These two levels are linked to the value of BDZs as reinforcers and thus, the question of reward (see Section 3.3 for possible issues with this interpretation). However, DSM criteria go further than this and include many maladaptive consequences of BDZ abuse, including the devaluation of natural reinforcers (e.g., food, sex) and giving these up in favor of BDZs, engaging in risky behaviors under the influence of or in order to acquire BDZs, and the neglect of responsibilities (e.g., poor parental behavior) due to BDZ abuse. As many of these behaviors may depend on the reinforcing value of the drug, with stronger reinforcers causing more maladaptive behaviors, we will be covering maladaptive behaviors under the general heading of reward-related behaviors. However, it should be noted that interactions with specific properties of drugs may influence each of these categories differentially. For instance, alcohol and stimulant use have different effects on the disinhibition of sexual behaviors and risk-taking (65).

At the level of simple preference, the preference of rodents for the midazolam-containing liquid in two-bottle choice experiments has been shown to depend on midazolam binding to α1 and α2GABAARs (24, 59). These studies employed mice with point mutations that make the targeted subunit insensitive to BDZs (14–16, 18, 19). While mice with point mutations on the α3 or α5 subunits continued to prefer midazolam-containing solution, this preference was abolished in mice with mutated α1 or α2 subunits.

In support of the integral role of α1 modulation in the pleasurable effects of BDZs, α1-preferring compound zolpidem is self-administered by non-human primates (NHPs) and has higher reinforcement value than non-selective BDZs, such as diazepam or midazolam, in self-administration tests (66–69). Comparison between zolpidem and midazolam is particularly relevant, as early studies suggest that short-acting BDZs act as stronger reinforcers than longer acting BDZs [(66, 70, 71); see (72) for a comparison of pharmacokinetic properties of commonly used BDZs]. Zolpidem, as a rapidly eliminated BDZ modulator, might owe its reinforcing value to its fast action as well as its receptor selectivity. Thus, a comparison with a rapidly eliminated non-selective BDZ, such as midazolam, isolates the role of receptor selectivity as a determinant of reinforcement value.

Self-administration of zolpidem demonstrates that α1-binding may be sufficient to sustain self-administration. Another relevant question is whether α1-binding is necessary. For instance, the 2-bottle choice experiments above indicate that α1-binding might be necessary for midazolam preference. Some studies (73) indeed suggest that α1-sparing compounds do not maintain self-administration in NHPs, agreeing with the necessity of α1-binding. Others (68, 69) suggest that sparing α1 is not sufficient to eliminate self-administration. Shinday et al. (69) elucidate the importance of drug history in this process, where α1-sparing compounds maintained self-administration in animals trained with midazolam, but not in animals trained with cocaine. As subjective stimulus properties of BDZs were shown to be primarily mediated by α1 in drug discrimination tests (67), this finding is unlikely to be a result of the subjective similarities between the effects of an α1-sparing drug and the training compound midazolam. Drug history was found to be important in the reinforcing effects of BDZs in humans as well, where non-selective BDZs were found to be more reinforcing in individuals with histories of sedative use and moderate alcohol consumption (34). It is possible that previous chronic exposure to GABAergic compounds causes changes in the expression and trafficking of GABAAR subtypes (74) and/or plasticity involving other systems (75, 76), such that α1-sparing compounds can activate brain circuitry involved in the experience of reward at a level that can maintain self-administration (see below for a more detailed discussion of plastic changes following long-term exposure to GABAergic drugs).

In cases where α1-sparing compounds are self-administered, efficacy at α2/3 seems critical for the maintenance of self-administration, based on reports that BDZ self-administration in NHPs is not influenced by the co-administration of an α5-selective negative modulator (77) and that compounds with reduced efficacy at α2/3 do not maintain self-administration (73). A role for α2GABAARs in reinforcing properties of BDZs has also been substantiated by ICSS studies in mice, where mice with mutated α2 subunits that render this subunit insensitive to the effects of BDZs no longer showed the reward-facilitating effects of diazepam or midazolam (24, 63). Similar to findings with self-administration of α1-sparing compounds by NHPs, Schwienteck et al. (78) reported that low-efficacy positive allosteric modulators with some selectivity for α2/3 lead to weak reward-facilitation in ICSS, suggesting that high-potency modulation of α2GABAARs might be both necessary and sufficient for self-administration and reward-facilitation effects. The demonstration of a role for α1GABAARs in ICSS has been less straightforward. While some studies suggested that α1-binding may be necessary (24) and sufficient (78), others noted negligible involvement of α1GABAARs in reward-facilitation effects (63). The differences in findings may be due to variability in dose ranges employed in different studies, as highly sedative compounds such as zolpidem can non-selectively reduce responding in ICSS giving the impression of reduced reward-facilitation, as well as to the variability in the drug histories of the animals in each study, as the studies involve sequential testing with multiple drugs. Finally, while the lack of α2- or α3-specific agents prevents conclusions regarding the individual contribution of each subtype to BDZ reward in pharmacological studies, the gene-targeted mouse studies suggest a possible involvement of α3GABAARs in the reward-enhancing effects of diazepam in ICSS (63), while such involvement was not found for reward-facilitation by midazolam (24), leaving the question of α3 involvement unresolved.

In summary, there is evidence that α1, α2, and possibly α3 subunits contribute to the reward-related effects of BDZs, with no involvement of α5GABAARs (77).

### 3.1. Maladaptive behaviors linked to BDZ use and GABAAR subtypes

The main maladaptive behaviors noted in DSM for Sedative, Hypnotic, and Anxiolytic Use Disorder can be categorized as those that represent abandoning natural rewards or responsibilities in favor of the drug and those that represent risky behaviors while using or to acquire the drug.

Devaluation of natural rewards (e.g., food, sex, caring for one's offspring, socializing) is a common consequence of drug addiction and has been investigated through animal models for different classes of drugs of abuse (79–83), often comparing drug responses to responses to palatable foods, such as sucrose. These experiments usually take the form of providing a sucrose solution while the animals are anticipating a drug reward. This leads to a comparison of the stronger drug reward with the now weaker, devalued natural reward. The effects of BDZs in this commonly used natural reward devaluation task have not been investigated. However, some early studies found a paradoxical role of drugs of abuse, including BDZs, in conditioned taste aversion (CTA) tasks (83). CTA tasks involve the pairing of a new, palatable food (e.g., a sucrose or saccharin solution) with an illness-inducing agent, such as lithium chloride. After this, animals avoid the consumption of the illness-associated stimulus. If the illness-associated stimulus is delivered intra-orally without operant behavior on the part of the animal, it is accompanied by suppressed ingestion responses, as well as active rejection responses such as gaping (84). The fact that preceding a palatable gustatory stimulus with a drug of abuse that is regularly self-administered by animals leads to reduced consumption of this stimulus was perplexing. Moreover, in the intra-oral delivery setting, the animals suppressed ingestion but showed no active rejection responses in this case, suggesting that the gustatory stimulus was not necessarily considered “aversive”. This type of suppression of response to natural reward has instead been considered a form of natural reward devaluation, where the animals show reduced interest in the natural stimulus that was previously linked with a BDZ or other drug of abuse, because the stimulus is now considered less rewarding (i.e., is devalued) compared to the greater reward of the drug (83). This reduction of interest in palatable gustatory stimuli due to BDZ pairing cannot be attributed to an aversive effect of BDZs, as these compounds are readily self-administered, or to an overall suppression of appetite, as BDZs are otherwise known to increase food intake (85), further supporting the likelihood of a natural reward devaluation due to reward comparison effect.

Caring for offspring can be conceived of as a natural reward and as a translational measure of carrying out responsibilities. While evidence suggests that acute or sub-chronic administration of BDZs causes impairments in maternal behavior and fragmented care for the offspring (86, 87), no studies to our knowledge investigated the question of maternal care in a free choice setting where the dams are provided with a choice to self-administer BDZs or care for offspring. As noted, studies also used acute or brief administration of BDZs which does not represent a drug use disorder scenario.

Overall, there is some support for the idea that BDZs might lead to devaluation of natural rewards, however, this question has not been systematically studied. Moreover, there is no information about the specific GABAAR subtypes that might be involved in this process to clarify whether the targeting of the specific GABAAR subtype might reduce the liability of natural reward devaluation compared to non-selective BDZs.

Acute administration of BDZs causes behavioral disinhibition and increased sensitivity to recent rewards, leading to increased risky decision-making (86, 88, 89). Strikingly, the facilitatory effects of BDZs on risky decision-making seemed limited to individuals with drug abuse histories and to relatively high doses of BDZs (89, 90), characteristics often observed in recreational BDZ users. Indeed, there is some evidence that polydrug users who also abuse BDZs engage in more risky behaviors compared to non-BDZ-using polydrug users (91, 92). Thus, there is some evidence that BDZ use may be associated with increased risk-taking behaviors, however, the brain mechanisms of BDZ-induced risk-taking are mostly unknown. One study showed that administration of lorazepam was linked to reduced activation of the amygdala and the medial prefrontal cortex and increased activation of the insular cortex during risky decision making [i.e., choosing of risky options over safe ones; (90)]. However, the study involved the administration of low doses of lorazepam which did not cause changes in risk-taking behaviors, which complicates the interpretation of the changes in brain activity. There have also been no studies to date investigating the involvement of different GABAAR subtypes in the promotion of risk-taking by BDZs. As all BDZ-sensitive GABAARs are expressed in the cortex and the amygdala, the findings from the Arce et al. (90) study also do not provide any clues as to which subtype(s) may be critical for the observed risk-promoting effects of BDZs. More relevant translationally is also the question of whether these acute effects are exacerbated upon chronic use, as is the case in the DSM definitions of Sedative, Hypnotic, and Anxiolytic Use Disorder, and how they might promote a cycle of risk-taking and drug use.

Overall, BDZs are self-administered and have been shown to facilitate reward effects in different species and a few studies investigated which GABAAR subtypes may be involved in these effects. However, maladaptive behavioral patterns observed in Sedative, Hypnotic, and Anxiolytic Use Disorder have not been studied in animal models, despite the availability of validated models from studies of other drugs of abuse. Thus, the question of whether specific GABAAR subtype(s) may play a central role in the progression of BDZ use from self-administration to a cycle of self-destructive behaviors remains open.

### 3.2. Brain mechanisms of BDZ reward

Drugs of abuse achieve their rewarding effects similarly to natural rewards, by increasing dopaminergic neurotransmission from the ventral tegmental area (VTA) to its mesolimbic target structures. While unexpected natural rewards initially cause increased dopamine firing in the VTA, after repeated presentation, the firing shifts to predictive cues from the reward itself (93). Importantly, drugs of abuse continue to cause increased firing even after repeated presentations, counter to the normal functioning of the brain reward system (60). Another important property of drugs of abuse is that they can induce long-lasting plasticity after even a single exposure (94). While the specific type of plasticity observed in the VTA depends on the mechanism of action of the specific drug of abuse, the overall effect is to cause increased dopamine release into the nucleus accumbens (NAc) and a priming of the VTA dopamine system that makes it more likely to respond to similar stimuli in the future.

BDZ actions on the mesocorticolimbic dopamine system are similar to other drugs of abuse. Specifically, BDZs increase dopamine release from the VTA onto target mesolimbic structures through a disinhibition mechanism, where BDZ binding to the GABAARs expressed on the VTA GABAergic interneurons leads to inhibition of the interneurons and the subsequent increased activation of the dopaminergic projection neurons (59, 60). Such a disinhibition-based mechanism is shared by some other drugs of abuse, such as opioids (95). In addition, like other drugs of abuse, a single injection of BDZs can cause VTA synaptic plasticity in the form of increased ratio of α-amino-3-hydroxy-5-methyl-4-isoxazolepropionic acid (AMPA) to *N*-methyl-D-aspartate (NMDA) receptor-mediated excitatory currents in the VTA for at least 3 days post-injection (96). BDZ binding to α1GABAARs seems to be both necessary and sufficient to induce BDZ-induced disinhibition and excitatory plasticity of the VTA dopamine neurons, as these effects were abolished in α1H101R mice that have BDZ-insensitive α1 subunits and the same effects could be induced by the α1-preferring GABAAR modulator zolpidem (59, 96). These physiological data provide a mechanistic explanation for the above behavioral findings noting self-administration of α1-preferring compounds and reduced ability of α1-sparing compounds to sustain self-administration.

Studies in rodents also point to the possibility of α2GABAAR involvement in BDZ-induced reward. α2GABAARs are expressed at negligible levels in the VTA, suggesting their involvement in BDZ reward may be through a different node in the brain reward system. Due to the high expression of α2GABAARs in the NAc, one possibility is that α2GABAARs mediate BDZ reward not by influencing dopamine release from VTA to target structures, but by modulating the effects of dopamine on those target structures such as the NAc. Viral-mediated knockdown of α2GABAARs in the NAc was indeed sufficient to abolish midazolam preference in a two-bottle choice drinking task (24). As α2GABAARs are expressed on both D1+ and D2+ medium spiny neurons (MSNs) of the NAc (97), it is difficult to speculate on an exact mechanism by which α2GABAARs of NAc regulate BDZ reward. Recent work suggests that α2GABAARs on D2+ NAc MSNs may be involved in the regulation of stress resiliency (29). As the effects of stress and subjective reward from BDZs seem to be closely linked (58, 98), it is possible that the α2GABAAR inhibitory regulation of D2+ MSNs plays a role in BDZ reward as well. Furthermore, α1GABAARs are expressed at high levels in the parvalbumin positive (PV+) interneurons of the NAc, which have been shown to play a significant role in motivated behaviors and the effects of drugs of abuse (99). The role of α1GABAARs in regulating the activity of this pivotal cell population indicates a second possible venue through which α1GABAARs might be involved in the reward-related effects of BDZs.

### 3.3. Issues related to interpretation and translation of findings from animal studies of BDZ reward

Studies using animal models provide a rich opportunity to understand pharmacological and brain mechanisms far beyond what could be achieved through studies in humans alone. However, like every modeling attempt, they come with certain possible confounds and alternative explanations that complicate the interpretation of findings within each model. Moreover, it is not clear whether findings from animal models can be directly translated to humans and caution should be exercised when drawing translational conclusions.

In animal models, the multitude of behavioral effects induced by BDZs often complicate the interpretation of results as purely reward-related. For instance, the two-bottle choice experiments where the rodents are presented with a bottle of water and a bottle of midazolam mixture may be affected by the sedative and amnestic effects of BDZs as well as their pleasurable subjective effects. Sedation may place a limit on drinking from the midazolam-containing bottle, as midazolam is fast-acting and highly sedative. The bottle placement is randomized every 24-h in these types of experiments, but amnestic effects may make it difficult for mice to learn which bottle has the pleasure-inducing liquid within the 24 h where the bottles remain put. Drugs affecting certain combinations of GABAARs may appear more preferred compared to other combinations due to increased pleasurable effects, or due to a reduction in sedative and/or amnestic effects, or when pleasurable effects and sedation and/or amnestic effects are mediated by the same receptor subtype, the pleasurable effects might be masked by the other effects. Similarly, while findings from the conditioned place preference test are often interpreted as drug-seeking behavior, they depend on the animal's ability to associate the context with the subjective effects of the drug during the training sessions and then retrieve this memory during the test session. Drugs with amnestic effects may interfere with this process. Drugs affecting specific receptor subtype combinations with reduced amnestic effects may look like they induce more drug-seeking behavior, purely due to better memory rather than increased reward, or again, reward-like effects might be masked by amnestic effects. ICSS, on the other hand, can be sensitive to the anticonvulsant effects of BDZs (100), as electrical stimulation of the forebrain can induce seizure activity. As anticonvulsant effects of BDZs are largely mediated by α1GABAARs (9), sparing binding to this subunit could increase ICSS thresholds (i.e., reduce apparent reward-facilitation by the compound) because of increased seizure susceptibility independent of any reward-related effects.

Self-administration studies often involve training with a drug that easily supports the acquisition of the operant behavior (e.g., cocaine), and then the ability of different drugs to maintain self-administration is tested. However, as noted in the above sections, for most drugs of abuse, even a single exposure can lead to long-lasting plastic effects in the brain reward circuitry. Moreover, we have noted that although there are points of convergence in the overall effects of drugs of abuse on the brain, the specific nature of these plastic effects depends on the properties of the drug. Based on this information, perhaps it is not surprising that Shinday et al. (69) found that the drug history of the animal determines whether an α1-sparing compound will maintain self-administration behavior or not. Thus, whether the animals received other compounds prior to testing and the specific properties of these compounds have the potential to affect outcomes and mask or supplement reward-related properties of the BDZs or subtype-selective compounds.

A final significant point is the comparability of the findings across species, and ultimately, the translatability of the findings to humans. Studies suggest many cross-species similarities in the expression of different GABAARs in brain areas relevant for the experience and processing of reward. For instance, high levels of α2 and α4, moderate-to-high levels of α1, and low-to-moderate levels of α3 expression in the striatum is observed in rodents (101–103), NHPs (104, 105), and humans (106). However, while α5 expression is undetectable in the striatum in rodents (101–103), studies report high levels of α5 in the NHP (105) and human (106) striatum. In the prefrontal cortex, while α5 expression is largely limited to layer 5, with low expression in other layers in rodents (101), the expression is more diffuse across layers in humans, with high expression in layers 4, 5 and 6, moderate expression in layers 2 and 3, and low expression in layer 1 (107). Thus, through strong expression in the striatum and more pronounced expression in the prefrontal cortex, α5 is more likely to have a role in reward processes in NHPs and humans than in rodents. In this sense, the finding that the co-administration of an α5-selective negative allosteric modulator did not influence triazolam self-administration in rhesus monkeys is highly relevant, suggesting that this subunit does not play an integral role in the maintenance of self-administration despite its dense expression in relevant brain areas in this species.

Based on the above-noted differences in GABAAR expression in different species, it is important to reemphasize here is that while all of the two-bottle choice, CPP, and ICSS studies reviewed above were conducted in rodents, all of the self-administration studies were conducted in NHPs. This adds another layer of complexity to comparative interpretation of the findings where differing task demands of different behavioral paradigms is also combined with possible species differences. Unfortunately, data on GABAAR expression in other relevant brain areas, such as the VTA, is missing in NHPs and humans, further adding to the uncertainty of the translatability of findings.

## 4. Tolerance to BDZ effects, BDZ withdrawal, and GABAAR subtypes

### 4.1. BDZ tolerance

Tolerance occurs at different rates for the different behavioral effects of BDZs, with rapid development of tolerance to the sedative and hypnotic effects, followed by the anticonvulsant effects (46, 57, 108–113). Tolerance to the anxiolytic effects is delayed and inconsistent in animal studies (114–118) and seems to be rare or non-existent in humans (46, 109, 110, 119, 120). Similarly, amnestic effects of BDZs do not seem to be attenuated during chronic treatment (111, 121–124). Lack of tolerance to amnestic effects can be considered a disadvantage, as amnestic effects are an undesirable side-effect of BDZs in most of their uses, particularly in case of elderly patients who take BDZs long-term, often for sleep problems (125–127).

A few studies addressed the question of whether the chronic modulation of specific GABAAR subtypes would lead to the same type of tolerance to specific behavioral effects as non-selective BDZs. Vinkers et al. (118) investigated the sedative, anxiolytic, and hypothermic effects of acute diazepam in mice treated chronically with diazepam, bretazenil [partial, non-selective GABAAR positive allosteric modulator (PAM)], zolpidem (α1-preferring PAM), or TPA023 (α2/3 preferring PAM). Tolerance was observed to all three effects in chronic dizepam treated animals. In bretazenil treated mice, cross-tolerance to anxiolytic and hypothermic effects were observed, although there was no tolerance to sedative effects. Most strikingly, zolpidem-treated mice showed full tolerance only to the hypothermic effects of diazepam, while no tolerance to any of the effects was observed in TPA023-treated mice.

At first sight, the finding that zolpidem did not lead to sedative tolerance is particularly surprising, as the sedative effects of BDZs are mediated primarily by the α1GABAARs, raising the expectation that sedative tolerance would also be observed with a compound that is selective for α1GABAARs. However, studies conducted on mice with mutations that render specific GABAAR subunits BDZ-insensitive indicate that BDZ-binding to α5GABAARs is required for the development of tolerance to the sedative effects of BDZs (128). In wild-type mice, the development of tolerance to the sedative effects of diazepam was associated with a decrease in the expression of α5 subunits in the dentate gyrus. In the context of these findings, lack of tolerance to zolpidem's sedative effects can be attributed to its lack of affinity for the α5GABAARs. In line with this, chronic treatment with a non-selective BDZ can cause cross-tolerance to the sedative effects of zolpidem (129), presumably due to the fact that chronic BDZ exposure has led to changes in dentate gyrus α5GABAAR expression during this time. Chronic BDZ treatment also causes a reduction in the expression of α1GABAARs in cortex (74, 130), which may also play a role in sedative tolerance.

Overall, studies suggest that α1-preferring compounds may cause little or no sedative tolerance compared to non-selective BDZs, however, findings are far from unequivocal (66, 74, 131–135). Moreover, as α1-preferring compounds are used long-term primarily for their hypnotic effects, the more clinically relevant question is whether tolerance develops to their hypnotic effects through chronic use. While some animal studies suggest that tolerance develops to the sleep-promoting effects of zolpidem over chronic administration (136), clinical work suggests less tolerance to the hypnotic effects of zolpidem compared to non-selective BDZs, at least at lower doses (137, 138).

Additionally, despite demonstrating tolerance to the anxiolytic-like properties of diazepam, the Vinkers et al. (118) study suggests that α2/3-selective compounds may provide anxiety relief even chronically, without any apparent tolerance to the anxiolytic effects. As α1-sparing compounds also do not cause sedation, this would be the ideal scenario for a long-term, effective anxiolytic. However, while previous studies suggested that α1GABAARs are required for the sedative effects of BDZs, recent preclinical work suggests that at high occupancy levels, BDZ binding to α3GABAARs may be sufficient to produce sedation (25). These preclinical findings also help to explain clinical findings that MK-409, a compound with selective efficacy at the α2/3GABAARs caused sedation in healthy volunteers (139).

The most relevant aspect of tolerance development to BDZ misuse and abuse would be the escalation of dose over use in order to attain the previous levels of pharmacological effect. However, studies show that escalation to higher doses over long-term use is rare with BDZs (44, 45, 140, 141). In summary, tolerance to specific effects of BDZs does not constitute a major problem from the perspective of BDZ misuse and there is some evidence that at least sedative tolerance can be circumvented through the use of α1GABAAR-selective compounds.

### 4.2. BDZ withdrawal

Tolerance and dependence are often viewed as related phenomena, both stemming from compensatory changes in the affected receptors and systems over prolonged exposure. However, experimental evidence suggests that the development of BDZ tolerance is not an indication that the individual will experience physical dependence to BDZs. On the contrary, BDZ tolerance and withdrawal seem to be independent phenomena where withdrawal symptoms can be observed in behavioral domains where no tolerance was observed and vice versa (64, 142). This behavioral distinction between tolerance and withdrawal is accompanied by distinct molecular effects of long-term exposure to BDZs vs. discontinuation of treatment [e.g., (143)].

Common BDZ withdrawal symptoms include agitation, anxiety, mood swings, muscle tension and spasms, feeling of “pins and needles”, perceptual sensitivity to light and sound, and seizures. Severe withdrawal can involve hallucinations and paranoid delusions, depersonalization, and can be fatal (57, 144, 145). Withdrawal symptoms appear within 2–3 days of cessation for short-acting BDZs and 5–10 days for longer-acting BDZs (137). Severe symptoms can mostly be avoided by gradual discontinuation over 6–8 weeks. However, even with managed discontinuation, it is estimated that up to half of the patients develop some level of withdrawal symptoms (145). For instance, in a study where patients were withdrawn from BDZs with individually calculated and managed withdrawal parameters over 2 weeks, with clinical monitoring every 48 h including physical examination and intensive psychological support and psychoeducation, 6 out of 9 long-term lorazepam users failed to discontinue the drug (57), demonstrating the significant challenge imposed by withdrawal symptoms to discontinuation of BDZs. Not surprisingly, particularly for patients who have been using BDZs long-term (i.e., more than 6 months) or at high doses (e.g., equivalent of 100 mg diazepam per day or more), hospitalization during the withdrawal period and pharmacological management of the symptoms is recommended (145, 146).

Overall, withdrawal symptoms upon BDZ discontinuation are common and serious. Withdrawal symptoms are the main driver of BDZ misuse and can contribute to abuse where users may start using BDZs primarily for their positive effects as outlined above, but are drawn into an abuse cycle as the primary motivator behind use switches to the avoidance of withdrawal symptoms (147).

Despite the clear significance of withdrawal symptoms and the availability of tools, such as gene-targeted mouse models and some pharmacological compounds with at least some selectivity for specific GABAAR subtypes, the role of specific GABAAR subtypes in BDZ withdrawal symptoms has been addressed in only a few studies. Work in NHPs has demonstrated withdrawal signs after the discontinuation of α1-preferring compounds and the recapitulation of flumazenil (non-selective BDZ antagonist) precipitated withdrawal by α1-selective antagonists (73, 113, 129). However, these studies included measurement of only a small subset of typical BDZ withdrawal symptoms and it is not clear whether α1-preferring agents might engender only a subset of withdrawal symptoms. Similarly, the duration or severity of withdrawal symptoms were not evaluated systematically in comparison to non-selective BDZs, leaving open the possibility that withdrawal from α1-preferring compounds might be milder, at least on certain symptoms, and/or briefer than that from non-selective BDZs. Finally, there is some evidence that discontinuation of α2/3-selective compounds may not result in a BDZ-like withdrawal syndrome (73, 129).

### 4.3. Brain mechanisms of BDZ tolerance and withdrawal

While it is tempting to assume that tolerance and withdrawal result simply from a compensatory mechanism whereby the cell-surface expression of the targeted receptor is reduced, BDZ tolerance and withdrawal seem to involve not only changes in GABAAR expression and function, but more complicated mechanisms that go beyond the GABAergic system.

Starting with the GABAergic changes, several studies reported changes in the expression levels of mRNAs for GABAAR receptor subunits upon chronic BDZ administration and discontinuation. As these changes have been thoroughly reviewed elsewhere (148) and seem to be complex and dependent on the brain area investigated, the specific BDZs employed, length and dose of administration, and whether the measures are taken at the end of the chronic administration period or following withdrawal, we will provide only a brief synopsis of the most common findings here.

The most common changes following chronic administration of BDZs are in expression of the α1 and α4 subunits (74, 149–154). While the findings have been mixed in terms of the presence of an effect, where effects were found, they were often in the direction of a reduction in α1 expression and an increase in α4 expression. Reduction in α1 expression in the cortex and the hippocampus has also been reported following withdrawal from chronic BDZs (124, 154). In experiments conducted in rat cerebellar granule cells, 5-day exposure of the cells to diazepam resulted in a decrease in α1 expression similar to the above *in vivo* studies (143). Withdrawal of diazepam, however, led to both a decrease in α1 and an increase in α4, suggesting discrete effects of chronic exposure and tolerance on GABAAR subunit expression. Withdrawal from zolpidem, an α1-preferring compound, led to similar changes in α1 and α4 expression as diazepam exposure *in vitro* (155). Similar reductions in α1 expression (in addition to α3 expression) were observed in the somatosensory cortex of mice following chronic exposure to zolpidem *in vivo* (74). An important conclusion of these findings is that the changes observed in GABAAR subunit expression are not limited to the subunits that are modulated by a given drug. We observe changes in the expression of the α4 subunit, which BDZs do not bind, following BDZ exposure and withdrawal, as well as changes in the α3 and α4 subunits following chronic exposure to an α1-preferring compound (74, 143, 155). Thus, for chronic exposure or withdrawal following a subtype-specific compound, we cannot assume that the GABAAR changes will be limited to the GABAAR subtype that is affected by this compound.

In addition to the above complex changes taking place in the GABAARs, BDZ tolerance and withdrawal involve other neurotransmitter systems in the brain. The glutamatergic system and synaptic plasticity involving NMDA and AMPA receptors, for instance, are causally involved in the development of a withdrawal syndrome following the cessation of chronic BDZ treatment (156). When the drug is withdrawn at the end of chronic BDZ treatment, there is often an asymptomatic refractory period of 3 to 5 days before the symptoms begin. Even for longest-acting BDZs, this refractory period is too long to be explained by the gradual clearance of the drug. During this refractory period, glutamatergic synapses go through a number of plastic changes with the insertion of AMPA receptors into the synapse and their subsequent phosphorylation, leading to increased AMPA/NMDA transmission ratio (157–163). Treatment with AMPA (but not NMDA) receptor antagonists during the refractory period abolishes the development of the withdrawal syndrome (164–167), demonstrating the causal involvement of this type of plasticity in excitatory synapses in the development of the withdrawal symptoms. A reduction in NMDA receptor expression and function is observed secondary to this enhancement of AMPA-mediated conductance (167) and the administration of NMDA receptor antagonists during the symptomatic portion of the withdrawal period can ameliorate symptoms (164). Even more strikingly, it was demonstrated that the co-administration of an NMDA receptor antagonist during chronic lorazepam administration can abolish tolerance to the anticonvulsant effects of lorazepam, although an overall reduction of BDZ-binding sites was observed in NMDA antagonist administered animals similar to controls (109), suggesting that glutamatergic mechanisms may be more important for the development of tolerance and dependence than changes in GABAAR expression.

The involvement of other systems and receptors [e.g., nitric oxide, (168); adenosine, (169); neuropeptide systems (170)] in the development of BDZ tolerance and/or withdrawal has been suggested, however, it is not clear whether the changes in these systems are essential for tolerance/dependence development or secondary to the observed changes in the glutamatergic and GABAergic systems.

Despite the well-established essential role of excitatory synaptic plasticity in the development of BDZ tolerance and withdrawal and close interactions between the glutamatergic and GABAergic systems, it is not known whether chronic modulation of specific GABAAR subtypes may lead to more rapid or enhanced glutamatergic plasticity. An understanding of these interactions would be essential for predicting dependence liability of subunit-specific GABAAR modulators. Similarly, it is not clear how GABAAR subtypes may interact with other neurotransmitter systems in a way that might exacerbate the observed tolerance and dependence symptoms, even if those neurotransmitter systems are not causally involved in the development of BDZ tolerance or BDZ withdrawal syndrome.

### 4.4. Issues related to interpretation and translation of findings from animal studies of BDZ tolerance and withdrawal

While hippocampal plasticity, which has been the focus of most studies related to BDZ withdrawal, is likely to be involved in the development of several withdrawal symptoms, it is highly likely that the development of tolerance to different behavioral effects of BDZs and the development of different withdrawal symptoms following BDZ discontinuation involve different brain areas. Similarly, different GABAAR subtypes might be involved in different withdrawal symptoms. Thus, behavioral studies covering all common withdrawal symptoms and systematically investigating the development of each following chronic modulation of a specific GABAAR subtype followed by drug discontinuation are needed. If only specific symptoms develop following discontinuation of a GABAAR subtype-specific modulation, this can also be used as an opportunity to study the brain mechanisms of specific withdrawal symptoms. The studies reviewed above, while informative, have not undertaken a detailed study of the withdrawal phenomenon and its mechanisms, and during a time other areas of neuroscience and neuropharmacology research have seen an explosion of new findings with unprecedented detail, our understanding of BDZ withdrawal has progressed relatively little since the early studies conducted in 1990s and early 2000s.

## 5. GABAARs in alcohol and other substance use disorders

GABAARs are expressed heavily in most brain regions involved in the effects of drugs of abuse and modulate the activity of brain circuits involved in the behavioral effects of drugs (171). As such, it is not surprising that different GABAARs have been implicated in the effects, use, and abuse of other drugs. Of these, alcohol is arguably the most relevant for discussion here due to its shared GABAergic mechanism.

Similar to BDZs, alcohol achieves most of its behavioral and subjective effects through positive allosteric modulation of GABAARs. Unlike BDZs, however, at high concentrations, alcohol modulates all GABAARs in an unselective manner, whereas at low concentrations (i.e., “social” drinking), synaptic GABAARs are mostly insensitive to alcohol's effects, whereas extrasynaptic, BDZ-insensitive GABAARs containing the δ subunits are highly sensitive to these low alcohol concentrations (172, 173). With chronic exposure, extrasynaptic responsiveness to ethanol decreases while synaptic responsiveness increases, with a concurrent relocation of α4GABAARs from extrasynaptic to synaptic locations (174). Changes in the expression and trafficking of other GABAARs, some of them similar to those observed with BDZ exposure, are also observed following chronic exposure to ethanol in animal models (175–178). In humans, several studies identified associations between GABRA2 gene (encoding the α2 subunit of the GABAAR) variations and alcohol use disorder (179–184). However, GABRA2 single nucleotide polymorphisms (SNPs) failed to reach significance on genome-wide association studies (GWAS) using more conservative analysis methods (185, 186). Still, GABRA2 gene expression was reduced in the hippocampi of alcohol dependent individuals in postmortem analyses (187). Others have found associations between polymorphisms in GABRA1 and GABRA6 genes and alcohol dependence (188, 189), however, again, these genes were not hits in GWAS studies.

Polymorphisms in the GABRA2 gene have also been implicated in stimulant (cocaine) and opioid (heroin) use disorders, particularly in interaction with early life adversity (190, 191). In cocaine-dependent individuals, GABRA2 SNPs were associated with cocaine cue reactivity (192). The involvement of α2GABAARs in some, but not all, effects of cocaine has also been confirmed in rodent studies (191, 193, 194). Finally, long-term exposure to cocaine was found to cause changes in the expression of α2GABAARs in the hippocampi of rodents (195), however, this finding was not confirmed in postmortem studies of hippocampi from individuals with cocaine use disorder (187). Others found that cocaine use disorder was associated with disruptions in several GABA-related genes in the postmortem dorsolateral prefrontal cortex, including GABRA1 and GABRA4. Interestingly, no changes were observed in genes related to glutamate signaling, emphasizing the special role of GABARs in the pathophysiology of substance use disorders (196).

## 6. Conclusions and directions

As seen, our knowledge regarding the involvement of specific GABAAR subtypes in all areas relevant to BDZ misuse and abuse, that is, reward processes, drug-related maladaptive behaviors, tolerance, and withdrawal, is characterized by gaps and a lack of systematic and mechanistic studies. Due to its central role in both BDZ misuse and BDZ abuse, an understanding of the mechanisms of BDZ withdrawal and how each GABAAR subtype is involved in the initiation and continuation of the withdrawal syndrome is particularly important. Research so far suggests that α1-sparing compounds would be highly desirable as anxiolytics, as they have the potential to provide anxiolysis without sedation and seem to have reduced abuse and misuse liability due to the apparent role of α1GABAARs in both the reward-related effects of BDZs and the development of a BDZ withdrawal syndrome upon cessation. However, some studies suggest the possible involvement of other GABAAR subtypes in these processes as well and it is not clear whether abolishing action at the α1GABAARs is sufficient to overcome potential for abuse and misuse. Considering the increasing burden of BDZ abuse, the common practice of BDZ misuse resulting in severe BDZ dependence in many patients, and the current efforts to produce subtype-specific GABAAR modulators as alternatives to classical BDZs, there is an urgent need for systematic and mechanistic research in this area.

## Author contributions

EE conducted the literature search and wrote the manuscript.

## References

[1] Food and Drug Administration. FDA requiring boxed warning updated to improve safe use of benzodiazepine drug class. (2020). Available online at: https://www.fda.gov/drugs/drug-safety-and-availability/fda-requiring-boxed-warning-updated-improve-safe-use-benzodiazepine-drug-class (accessed November 11, 2022).

[2] Alonso J Angermeyer MC Bernert S Bruffaerts R Brugha TS Bryson H. Psychotropic drug utilization in Europe: results from the European Study of the Epidemiology of Mental Disorders (ESEMeD) project. Acta Psychiatr Scand Suppl. (2004) 109:55–64. 10.1111/j.1600-0047.2004.00325.x 15128388

[3] Huerta C Abbing-Karahagopian V Requena G Oliva B Alvarez Y Gardarsdottir H. Exposure to benzodiazepines (anxiolytics, hypnotics and related drugs) in seven European electronic healthcare databases: a cross-national descriptive study from the PROTECT-EU Project. Pharmacoepidemiol Drug Saf. (2016) 25:56–65. 10.1002/pds.3825 26149383

[4] United Nations Office on Drugs and Crime. Non-medical use of benzodiazepines: A growing threat to public health? Global Smart Update. (2017) 18.

[5] Hockenhull J Amioka E Black JC Haynes CM Dargan PI Dart RC. Nonmedical use of alprazolam in the UK: Results from a nationally representative survey. Br J Clin Pharmacol. (2019) 85:1841–5. 10.1111/bcp.13959 31165490 PMC6624393

[6] Hockenhull J Black JC Haynes CM Rockhill K Dargan PI Dart RC. Nonmedical use of benzodiazepines and Z-drugs in the UK. Br J Clin Pharmacol. (2021) 87:1676–83. 10.1111/bcp.14397 32472941

[7] Hockenhull J Amioka E Black JC Forber A Haynes CM Wood DM. Non-medical use of benzodiazepines and GABA analogues in Europe. Br J Clin Pharmacol. (2021) 87:1684–94. 10.1111/bcp.14537 32888191

[8] Zheng D Brett J Daniels B Buckley NA Pearson SA Schaffer AL. Potentially inappropriate benzodiazepine use in Australian adults: A population-based study (2014-2017). Drug Alcohol Rev. (2020) 39:575–82. 10.1111/dar.13086 32391624

[9] Rudolph U Knoflach F. Beyond classical benzodiazepines: novel therapeutic potential of GABA(A) receptor subtypes. Nat Rev Drug Discor. (2011) 10:685–97. 10.1038/nrd3502 21799515 PMC3375401

[10] Chua HC Chebib M. GABAA Receptors and the diversity in their structure and pharmacology. Adv Pharmacol. (2017) 79:1–34. 10.1016/bs.apha.2017.03.003 28528665

[11] Engin E Benham RS Rudolph U. An emerging circuit pharmacology of GABA. Trends Pharmacol Sci. (2018) 39:710–32. 10.1016/j.tips.2018.04.003 29903580 PMC6056379

[12] Scott S Aricescu AR. A structural perspective on GABA. Curr Opin Struct Biol. (2019) 54:189–97. 10.1016/j.sbi.2019.03.023 31129381

[13] Liu R Wang J Liang S Zhang G Yang X. Role of NKCC1 and KCC2 in Epilepsy: From Expression to Function. Front Neurol. (2020) 10:1407. 10.3389/fneur.2019.01407 32010056 PMC6978738

[14] Rudolph U Crestani F Benke D Brunig I Benson JA Fritschy JM. Benzodiazepine actions mediated by specific gamma-aminobutyric acid(A) receptor subtypes. Nature. (1999) 401:796–800. 10.1038/44579 10548105

[15] Löw K Crestani F Keist R Benke D Brünig I Benson JA. Molecular and neuronal substrate for the selective attenuation of anxiety. Science. (2000) 290:131–4. 10.1126/science.290.5489.131 11021797

[16] Crestani F Assandri R Tauber M Martin JR Rudolph U. Contribution of the alpha1-GABA(A) receptor subtype to the pharmacological actions of benzodiazepine site inverse agonists. Neuropharmacology. (2002) 43:679–84. 10.1016/S0028-3908(02)00159-4 12367613

[17] Collinson N Kuenzi FM Jarolimek W Maubach KA Cothliff R Sur C. Enhanced learning and memory and altered GABAergic synaptic transmission in mice lacking the alpha 5 subunit of the GABAA receptor. J Neurosci. (2002) 22:5572–80. 10.1523/JNEUROSCI.22-13-05572.2002 12097508 PMC6758233

[18] Yee BK Keist R von Boehmer L Studer R Benke D Hagenbuch N. A schizophrenia-related sensorimotor deficit links alpha 3-containing GABAA receptors to a dopamine hyperfunction. Proc Natl Acad Sci U S A. (2005) 102:17154–9. 10.1073/pnas.0508752102 16284244 PMC1288020

[19] Yee BK Hauser J Dolgov VV Keist R Mohler H Rudolph U. GABA receptors containing the alpha5 subunit mediate the trace effect in aversive and appetitive conditioning and extinction of conditioned fear. Eur J Neuroscie. (2004) 20:1928–36. 10.1111/j.1460-9568.2004.03642.x 15380015

[20] Smith KS Engin E Meloni EG Rudolph U. Benzodiazepine-induced anxiolysis and reduction of conditioned fear are mediated by distinct GABAA receptor subtypes in mice. Neuropharmacology. (2012) 63:250–8. 10.1016/j.neuropharm.2012.03.001 22465203 PMC3372637

[21] Engin E Zarnowska ED Benke D Tsvetkov E Sigal M Keist R. Tonic inhibitory control of dentate gyrus granule cells by alpha5-containing GABAA receptors reduces memory interference. J Neuroscience. (2015) 35:13698–712. 10.1523/JNEUROSCI.1370-15.2015 26446222 PMC4595621

[22] Engin E Smith KS Gao Y Nagy D Foster RA Tsvetkov E. Modulation of anxiety and fear via distinct intrahippocampal circuits. Elife. (2016) 5:e14120. 10.7554/eLife.14120 26971710 PMC4816644

[23] Engin E Sigal M Benke D Zeller A Rudolph U. Bidirectional regulation of distinct memory domains by α5-subunit-containing GABA. Learn Mem. (2020) 27:423–8. 10.1101/lm.052084.120 32934095 PMC7497110

[24] Engin E Bakhurin KI Smith KS Hines RM Reynolds LM Tang W. Neural basis of benzodiazepine reward: requirement for alpha2 containing GABAA receptors in the nucleus accumbens. Neuropsychopharmacology. (2014) 39:1805–15. 10.1038/npp.2014.41 24553732 PMC4059902

[25] Behlke LM Foster RA Liu J Benke D Benham RS Nathanson AJ. A pharmacogenetic 'restriction-of-function' approach reveals evidence for anxiolytic-like actions mediated by alpha5-containing GABAA receptors in mice. Neuropsychopharmacology. (2016) 41:2492–501. 10.1038/npp.2016.49 27067130 PMC4987847

[26] Zeilhofer HU Möhler H Di Lio A. GABAergic analgesia: new insights from mutant mice and subtype-selective agonists. Trends Pharmacol Sci. (2009) 30:397–402. 10.1016/j.tips.2009.05.007 19616317

[27] Ralvenius WT Benke D Acuna MA Rudolph U Zeilhofer HU. Analgesia and unwanted benzodiazepine effects in point-mutated mice expressing only one benzodiazepine-sensitive GABA(A) receptor subtype. Nat Commun. (2015) 6:6803. 10.1038/ncomms7803 25865415 PMC4829939

[28] Prévot T Sibille E. Altered GABA-mediated information processing and cognitive dysfunctions in depression and other brain disorders. Mol Psychiatry. (2021) 26:151–67. 10.1038/s41380-020-0727-3 32346158

[29] Benham RS Choi C Hodgson NW Hewage NB Kastli R Donahue RJ. α2-containing γ-aminobutyric acid type A receptors promote stress resiliency in male mice. Neuropsychopharmacology. (2021) 46:2197–206. 10.1038/s41386-021-01144-w 34408277 PMC8505491

[30] Bernardo A Lee P Marcotte M Mian MY Rezvanian S Sharmin D. Symptomatic and neurotrophic effects of GABAA receptor positive allosteric modulation in a mouse model of chronic stress. Neuropsychopharmacology. (2022) 47:1608–19. 10.1038/s41386-022-01360-y 35701547 PMC9283409

[31] Sieghart W Savić MM. International Union of Basic and Clinical Pharmacology. CVI: GABAA receptor subtype- and function-selective ligands: key issues in translation to humans. Pharmacol Rev. (2018) 70:836–78. 10.1124/pr.117.014449 30275042

[32] Maramai S Benchekroun M Ward SE Atack JR. Subtype selective γ-aminobutyric acid type a receptor (GABAAR) modulators acting at the benzodiazepine binding site: an update. J Med Chem. (2020) 63:3425–46. 10.1021/acs.jmedchem.9b01312 31738537

[33] Association AP. Diagnostic and Statistical Manual of Mental Disorders. 5th Edition, Text Revision ed. (2022).

[34] Griffiths RR Weerts EM. Benzodiazepine self-administration in humans and laboratory animals–implications for problems of long-term use and abuse. Psychopharmacology (Berl). (1997) 134:1–37. 10.1007/s002130050422 9399364

[35] O'brien CP. Benzodiazepine use, abuse, and dependence. J Clin Psychiatry. (2005) 66:28–33. 15762817

[36] Jones JD Mogali S Comer SD. Polydrug abuse: a review of opioid and benzodiazepine combination use. Drug Alcohol Depend. (2012) 125:8–18. 10.1016/j.drugalcdep.2012.07.004 22857878 PMC3454351

[37] Zamboni L Portoghese I Congiu A Zandonai T Casari R Fusina F. Polysubstance use patterns among high dose benzodiazepine users: a latent class analysis and differences between male and female use. Front Psychiatry. (2022) 13:811130. 10.3389/fpsyt.2022.811130 35145442 PMC8821140

[38] Moosmann B King LA Auwärter V. Designer benzodiazepines: A new challenge. World Psychiatry. (2015) 14:248. 10.1002/wps.20236 26043347 PMC4471986

[39] Liebrenz M Schneider M Buadze A Gehring MT Dube A Caflisch C. High-dose benzodiazepine dependence: a qualitative study of patients' perceptions on initiation, reasons for use, and obtainment. PLoS ONE. (2015) 10:e0142057. 10.1371/journal.pone.0142057 26556055 PMC4640837

[40] Kharadi D Patel K Rana D Patel V. Off-label drug use in Psychiatry Outpatient Department: A prospective study at a Tertiary Care Teaching Hospital. J Basic Clin Pharm. (2015) 6:45–9. 10.4103/0976-0105.152090 25767363 PMC4356999

[41] López-Pelayo H Coma A Gual A Zara C Lligoña A. Call for action: benzodiazepine prescription prevalence analysis shows off-label prescription in one in eleven citizens. Eur Addict Res. (2019) 25:320–9. 10.1159/000502518 31494655

[42] Lücke C Gschossmann JM Grömer TW Moeller S Schneider CE Zikidi A. Off-label prescription of psychiatric drugs by non-psychiatrist physicians in three general hospitals in Germany. Ann Gen Psychiatry. (2018) 17:7. 10.1186/s12991-018-0176-4 29449869 PMC5806239

[43] Vijay A Becker JE Ross JS. Patterns and predictors of off-label prescription of psychiatric drugs. PLoS ONE. (2018) 13:e0198363. 10.1371/journal.pone.0198363 30024873 PMC6053129

[44] Alessi-Severini S Bolton JM Enns MW Dahl ME Chateau D Collins DM. Sustained use of benzodiazepines and escalation to high doses in a Canadian population. Psychiatr Serv. (2016) 67:1012–8. 10.1176/appi.ps.201500380 27133727

[45] Willems IA Gorgels WJ Oude Voshaar RC Mulder J Lucassen PL. Tolerance to benzodiazepines among long-term users in primary care. Fam Pract. (2013) 30:404–10. 10.1093/fampra/cmt010 23515374

[46] Bateson AN. Basic pharmacologic mechanisms involved in benzodiazepine tolerance and withdrawal. Curr Pharm Des. (2002) 8:5–21. 10.2174/1381612023396681 11812247

[47] Barter G Cormack M. The long-term use of benzodiazepines: patients' views, accounts and experiences. Fam Pract. (1996) 13:491–7. 10.1093/fampra/13.6.491 9023523

[48] Ashton H. The diagnosis and management of benzodiazepine dependence. Curr Opin Psychiatry. (2005) 18:249–55. 10.1097/01.yco.0000165594.60434.84 16639148

[49] Donoghue J Lader M. Usage of benzodiazepines: A review. Int J Psychiatry Clin Pract. (2010) 14:78–87. 10.3109/13651500903447810 24922466

[50] Olfson M King M Schoenbaum M. Benzodiazepine use in the United States. JAMA Psychiatry. (2015) 72:136–42. 10.1001/jamapsychiatry.2014.1763 25517224

[51] Lader M. Benzodiazepines revisited–will we ever learn? Addiction. (2011) 106:2086–109. 10.1111/j.1360-0443.2011.03563.x 21714826

[52] Baldwin DS Aitchison K Bateson A Curran HV Davies S Leonard B. Benzodiazepines: risks and benefits. A reconsideration. J Psychopharmacol. (2013) 27:967–71. 10.1177/0269881113503509 24067791

[53] Baldwin DS. Clinical management of withdrawal from benzodiazepine anxiolytic and hypnotic medications. Addiction. (2022) 117:1472–82. 10.1111/add.15695 34542216

[54] Maust DT Lin LA Blow FC. Benzodiazepine use and misuse among adults in the United States. Psychiatr Serv. (2019) 70:97–106. 10.1176/appi.ps.201800321 30554562 PMC6358464

[55] Finlayson AJR Macoubrie J Huff C Foster DE Martin PR. Experiences with benzodiazepine use, tapering, and discontinuation: an Internet survery. Therap Adv Psychopharmacol. (2022) 12:1–10. 10.1177/20451253221082386 35499041 PMC9047812

[56] O'Connor KP Marchand A Bélanger L Mainguy N Landry P Savard P. Psychological distress and adaptational problems associated with benzodiazepine withdrawal and outcome: a replication. Addict Behav. (2004) 29:583–93. 10.1016/j.addbeh.2004.01.001 15050676

[57] Poyares D Guilleminault C Ohayon MM Tufik S. Chronic benzodiazepine usage and withdrawal in insomnia patients. J Psychiatr Res. (2004) 38:327–34. 10.1016/j.jpsychires.2003.10.003 15003439

[58] Sherwin C Olsson I. Housing conditions affect self-administration of anxiolytic by laboratory mice. Animal Welfare. (2004) 13:33–8.17018210

[59] Tan KR Brown M Labouebe G Yvon C Creton C Fritschy JM. Neural bases for addictive properties of benzodiazepines. Nature. (2010) 463:769–74. 10.1038/nature08758 20148031 PMC2871668

[60] Tan KR Rudolph U Luscher C. Hooked on benzodiazepines: GABAA receptor subtypes and addiction. Trends Neurosci. (2011) 34:188–97. 10.1016/j.tins.2011.01.004 21353710 PMC4020178

[61] Spyraki C Kazandjian A Varonos D. Diazepam-induced place preference conditioning: appetitive and antiaversive properties. Psychopharmacology (Berl). (1985) 87:225–32. 10.1007/BF00431813 3931151

[62] Licata SC Rowlett JK. Abuse and dependence liability of benzodiazepine-type drugs: GABA(A) receptor modulation and beyond. Pharmacol Biochem Behav. (2008) 90:74–89. 10.1016/j.pbb.2008.01.001 18295321 PMC2453238

[63] Reynolds LM Engin E Tantillo G Lau HM Muschamp JW Carlezon WA. Differential roles of GABA(A) receptor subtypes in benzodiazepine-induced enhancement of brain-stimulation reward. Neuropsychopharmacology. (2012) 37:2531–40. 10.1038/npp.2012.115 22763624 PMC3442348

[64] Moerke MJ Li G Golani LK Cook J Negus SS. Effects of the α2/α3-subtype-selective GABAA receptor positive allosteric modulator KRM-II-81 on pain-depressed behavior in rats: comparison with ketorolac and diazepam. Behav Pharmacol. (2019) 30:452–61. 10.1097/FBP.0000000000000464 30640180 PMC6610697

[65] Frohmader KS Pitchers KK Balfour ME Coolen LM. Mixing pleasures: review of the effects of drugs on sex behavior in humans and animal models. Horm Behav. (2010) 58:149–62. 10.1016/j.yhbeh.2009.11.009 20004662

[66] Griffiths RR Sannerud CA Ator NA Brady JV. Zolpidem behavioral pharmacology in baboons: self-injection, discrimination, tolerance and withdrawal. J Pharmacol Exp Ther. (1992) 260:1199–208. 1312162

[67] Rowlett JK Platt DM Lelas S Atack JR Dawson GR. Different GABAA receptor subtypes mediate the anxiolytic, abuse-related, and motor effects of benzodiazepine-like drugs in primates. Proc Natl Acad Sci U S A. (2005) 102:915–20. 10.1073/pnas.0405621102 15644443 PMC545524

[68] Rowlett JK Lelas S. Comparison of zolpidem and midazolam self-administration under progressive-ratio schedules: consumer demand and labor supply analyses. Exp Clin Psychopharmacol. (2007) 15:328–37. 10.1037/1064-1297.15.4.328 17696679

[69] Shinday NM Sawyer EK Fischer BD Platt DM Licata SC Atack JR. Reinforcing effects of compounds lacking intrinsic efficacy at α1 subunit-containing GABAA receptor subtypes in midazolam- but not cocaine-experienced rhesus monkeys. Neuropsychopharmacology. (2013) 38:1006–14. 10.1038/npp.2012.265 23303046 PMC3629390

[70] Griffiths RR Bigelow GE Liebson I. Human preference comparison of pentobarbital, diazepam, and placebo. NIDA Res Monogr. (1981) 34:220–5. 6783936

[71] Griffiths RR Lamb RJ Sannerud CA Ator NA Brady JV. Self-injection of barbiturates, benzodiazepines and other sedative-anxiolytics in baboons. Psychopharmacology (Berl). (1991) 103:154–61. 10.1007/BF02244196 1674158

[72] Peng L Morford K. L, Laevander X. A benzodiazepines and related sedatives. Med Clin North America. (2022) 106:113–29. 10.1016/j.mcna.2021.08.012 34823725

[73] Ator NA Atack JR Hargreaves RJ Burns HD Dawson GR. Reducing abuse liability of GABAA/benzodiazepine ligands via selective partial agonist efficacy at alpha1 and alpha2/3 subtypes. J Pharmacol Exp Ther. (2010) 332:4–16. 10.1124/jpet.109.158303 19789360 PMC2802472

[74] Wright BT Gluszek CF Heldt SA. The effects of repeated zolpidem treatment on tolerance, withdrawal-like symptoms, and GABAA receptor mRNAs profile expression in mice: Comparison with diazepam. Psychopharmacology. (2014) 231:2967–79. 10.1007/s00213-014-3473-x 24531568

[75] Xiang K Tietz EI. Benzodiazepine-induced hippocampal CA1 neuron alpha-amino-3-hydroxy-5-methylisoxasole-4-propionic acid (AMPA) receptor plasticity linked to severity of withdrawal anxiety: differential role of voltage-gated calcium channels and N-methyl-D-aspartic acid receptors. Behav Pharmacol. (2007) 18:447–60. 10.1097/FBP.0b013e3282d28f2b 17762513

[76] Monti MC Almiron RS Bignante EA Ramirez OA. Changes in hippocampal arc protein expression and synaptic plasticity by the presentation of contextual cues linked to drug experience. Synapse. (2010) 64:39–46. 10.1002/syn.20700 19728364

[77] Fischer BD Platt DM Rallapalli SK Namjoshi OA Cook JM Rowlett JK. Antagonism of triazolam self-administration in rhesus monkeys responding under a progressive-ratio schedule: In vivo apparent pA2 analysis. Drug Alcohol Depend. (2016) 158:22–9. 10.1016/j.drugalcdep.2015.10.026 26596587 PMC4698084

[78] Schwienteck KL Li G Poe MM Cook JM Banks ML Stevens Negus S. Abuse-related effects of subtype-selective GABA. Psychopharmacology (Berl). (2017) 234:2091–101. 10.1007/s00213-017-4615-8 28365836 PMC5875719

[79] Moschak TM Wang X Carelli RM A. Neuronal ensemble in the rostral agranular insula tracks cocaine-induced devaluation of natural reward and predicts cocaine seeking. J Neurosci. (2018) 38:8463–72. 10.1523/JNEUROSCI.1195-18.2018 30126972 PMC6158695

[80] Grigson PS Twining RC. Cocaine-induced suppression of saccharin intake: a model of drug-induced devaluation of natural rewards. Behav Neurosci. (2002) 116:321–33. 10.1037/0735-7044.116.2.321 11996317

[81] Freet CS Alexander DN Imperio CG Ruiz-Velasco V Grigson PS. Heroin-induced suppression of saccharin intake in OPRM1 A118G mice. Brain Res Bull. (2018) 138:73–9. 10.1016/j.brainresbull.2017.09.008 28939474 PMC5860935

[82] McFalls AJ Imperio CG Bixler G Freeman WM Grigson PS Vrana KE. Reward devaluation and heroin escalation is associated with differential expression of CRF signaling genes. Brain Res Bull. (2016) 123:81–93. 10.1016/j.brainresbull.2015.11.009 26655889

[83] Grigson PS. Reward Comparison: The Achilles' heel and hope for addiction. Drug Discov Today Dis Models. (2008) 5:227–33. 10.1016/j.ddmod.2009.03.005 20016772 PMC2794208

[84] Parker LA. Rewarding drugs produce taste avoidance, but not taste aversion. Neurosci Biobehav Rev. (1995) 19:143–57. 10.1016/0149-7634(94)00028-Y 7770194

[85] Berridge KC Pecina S. Benzodiazepines, appetite, and taste palatability. Neurosci Biobehav Rev. (1995) 19:121–31. 10.1016/0149-7634(94)00026-W 7770192

[86] Laviola G Bignami G Alleva E. Interacting effects of oxazepam in late pregnancy and fostering procedure on mouse maternal behavior. Neurosci Biobehav Rev. (1991) 15:501–4. 10.1016/S0149-7634(05)80139-3 1792012

[87] Ferreira A Picazo O Uriarte N Pereira M Fernández-Guasti A. Inhibitory effect of buspirone and diazepam, but not of 8-OH-DPAT, on maternal behavior and aggression. Pharmacol Biochem Behav. (2000) 66:389–96. 10.1016/S0091-3057(00)00211-2 10880695

[88] Lane SD Cherek DR Nouvion SO. Modulation of human risky decision making by flunitrazepam. Psychopharmacology (Berl). (2008) 196:177–88. 10.1007/s00213-007-0951-4 17917718

[89] Lane SD Tcheremissine OV Lieving LM Nouvion S Cherek DR. Acute effects of alprazolam on risky decision making in humans. Psychopharmacology (Berl). (2005) 181:364–73. 10.1007/s00213-005-2265-8 15830221

[90] Arce E Miller DA Feinstein JS Stein MB Paulus MP. Lorazepam dose-dependently decreases risk-taking related activation in limbic areas. Psychopharmacology (Berl). (2006) 189:105–16. 10.1007/s00213-006-0519-8 17016713 PMC2839080

[91] Darke S Hall W Ross M Wodak A. Benzodiazepine use and HIV risk-taking behaviour among injecting drug users. Drug Alcohol Depend. (1992) 31:31–6. 10.1016/0376-8716(92)90005-W 1358587

[92] Caplehorn JR Saunders JB. Factors associated with heroin users' AIDS risk-taking behaviours. Aust J Public Health. (1993) 17:13–7. 10.1111/j.1753-6405.1993.tb00097.x 8357887

[93] Schultz W. Dopamine signals for reward value and risk: basic and recent data. Behav Brain Functions. (2010) 6:24–32. 10.1186/1744-9081-6-24 20416052 PMC2876988

[94] Volkow ND Michaelides M Baler R. The neuroscience of drug reward and addiction. Physiol Rev. (2019) 99:2115–40. 10.1152/physrev.00014.2018 31507244 PMC6890985

[95] Johnson SW North RA. Opioids excite dopamine neurons by hyperpolarization of local interneurons. J Neurosci. (1992) 12:483–8. 10.1523/JNEUROSCI.12-02-00483.1992 1346804 PMC6575608

[96] Heikkinen AE Moykkynen TP Korpi ER. Long-lasting modulation of glutamatergic transmission in VTA dopamine neurons after a single dose of benzodiazepine agonists. Neuropsychopharmacology. (2009) 34:290–8. 10.1038/npp.2008.89 18563060

[97] Saunders A Macosko EZ Wysoker A Goldman M Krienen F de Rivera H. Molecular diversity and specializations among the cells of the adult mouse brain. Cell. (2018) 174:1015–30. 10.1016/j.cell.2018.07.028 30096299 PMC6447408

[98] Wichniak A Brunner H Ising M Pedrosa Gil F Holsboer F Friess E. Impaired hypothalamic-pituitary-adrenocortical (HPA) system is related to severity of benzodiazepine withdrawal in patients with depression. Psychoneuroendocrinology. (2004) 29:1101–8. 10.1016/j.psyneuen.2003.11.004 15219633

[99] Schall TA Wright WJ Dong Y. Nucleus accumbens fast-spiking interneurons in motivational and addictive behaviors. Molecular Psychiatry. (2020) 26:234–46. 10.1038/s41380-020-0683-y 32071384 PMC7431371

[100] Bielajew CH Harris T. Self-stimulation: A rewarding decade. J Psychiat Neurosci. (1991) 16:109–14. PMC1188317 1958643

[101] Fritschy JM Mohler H. GABAA-receptor heterogeneity in the adult rat brain: differential regional and cellular distribution of seven major subunits. J Compar Neurol. (1995) 359:154–94. 10.1002/cne.903590111 8557845

[102] Hörtnagl H Tasan RO Wieselthaler A Kirchmair E Sieghart W Sperk G. Patterns of mRNA and protein expression for 12 GABAA receptor subunits in the mouse brain. Neuroscience. (2013) 236:345–72. 10.1016/j.neuroscience.2013.01.008 23337532 PMC3605588

[103] Pirker S Schwarzer C Wieselthaler A Sieghart W Sperk G. GABA(A) receptors: immunocytochemical distribution of 13 subunits in the adult rat brain. Neuroscience. (2000) 101:815–50. 10.1016/S0306-4522(00)00442-5 11113332

[104] Kultas-Ilinsky K Leontiev V Whiting PJ. Expression of 10 GABA(A) receptor subunit messenger RNAs in the motor-related thalamic nuclei and basal ganglia of Macaca mulatta studied with in situ hybridization histochemistry. Neuroscience. (1998) 85:179–204. 10.1016/S0306-4522(97)00634-9 9607711

[105] Sperk G Kirchmair E Bakker J Sieghart W Drexel M Kondova I. Immunohistochemical distribution of 10 GABA(A) receptor subunits in the forebrain of the rhesus monkey Macaca mulatta. J Comp Neurol. (2020) 528:2551–68. 10.1002/cne.24910 32220012 PMC7496627

[106] Bhandage AK Jin Z Bazov I Kononenko O Bakalkin G Korpi ER. GABA-A and NMDA receptor subunit mRNA expression is altered in the caudate but not the putamen of the postmortem brains of alcoholics. Front Cell Neurosci. (2014) 8:415. 10.3389/fncel.2014.00415 25538565 PMC4257153

[107] Akbarian S Huntsman MM Kim JJ Tafazzoli A Potkin SG Bunney WE. GABAA receptor subunit gene expression in human prefrontal cortex: comparison of schizophrenics and controls. Cereb Cortex. (1995) 5:550–60. 10.1093/cercor/5.6.550 8590827

[108] Galpern WR Lumpkin M Greenblatt DJ Shader RI Miller LG. Chronic benzodiazepine administration. VII Behavioral tolerance and withdrawal and receptor alterations associated with clonazepam administration. Psychopharmacology (Berl). (1991) 104:225–30. 10.1007/BF02244183 1652144

[109] Koff JM Pritchard GA Greenblatt DJ Miller LG. The NMDA receptor competitive antagonist CPP modulates benzodiazepine tolerance and discontinuation. Pharmacology. (1997) 55:217–27. 10.1159/000139531 9399331

[110] Pratt JA Brett RR Laurie DJ. Benzodiazepine dependence: from neural circuits to gene expression. Pharmacol Biochem Behav. (1998) 59:925–34. 10.1016/S0091-3057(97)00539-X 9586850

[111] Vinkers CH Olivier B. Mechanisms underlying tolerance after long-term benzodiazepine use: a future for subtype-selective GABA(A) receptor modulators? Adv Pharmacol Sci. (2012) 2012:416864. 10.1155/2012/416864 22536226 PMC3321276

[112] Gravielle MC. Activation-induced regulation of GABAA receptors: Is there a link with the molecular basis of benzodiazepine tolerance? Pharmacol Res. (2016) 109:92–100. 10.1016/j.phrs.2015.12.030 26733466

[113] Duke AN Platt DM Rowlett JK. Tolerance and dependence following chronic alprazolam treatment: quantitative observation studies in female rhesus monkeys. Psychopharmacology (Berl). (2020) 237:1183–94. 10.1007/s00213-019-05447-1 31927603 PMC7988478

[114] File SE. Tolerance to the behavioral actions of benzodiazepines. Neurosci Biobehav Rev. (1985) 9:113–21. 10.1016/0149-7634(85)90037-5 2858076

[115] Ishihara S Hiramatsu M Kameyama T Nabeshima T. Development of tolerance to amnesic effects of chlordiazepoxide in relation to GABAergic and cholinergic neuronal systems. Eur J Pharmacol. (1993) 230:313–20. 10.1016/0014-2999(93)90567-2 8382617

[116] Kita A Kinoshita T Kohayakawa H Furukawa K Akaike A. Lack of tolerance to anxiolysis and withdrawal symptoms in mice repeatedly treated with AC-5216, a selective TSPO ligand. Prog Neuropsychopharmacol Biol Psychiatry. (2009) 33:1040–5. 10.1016/j.pnpbp.2009.05.018 19497344

[117] Ferreri MC Gutierrez ML Gravielle MC. Tolerance to the sedative and anxiolytic effects of diazepam is associated with different alterations of GABAA receptors in rat cerebral cortex. Neuroscience. (2015) 310:152–62. 10.1016/j.neuroscience.2015.09.038 26391922

[118] Vinkers CH van Oorschot R Nielsen EO Cook JM Hansen HH Groenink L. GABAA receptor a subunits differentially contribute to diazepam tolerance after chronic treatment. PLoS ONE. (2012) 7:e43054. 10.1371/journal.pone.0043054 22912786 PMC3418228

[119] Rickels K Case WG Downing RW Winokur A. Long-term diazepam therapy and clinical outcome. JAMA. (1983) 250:767–71. 10.1001/jama.250.6.767 6348314

[120] Ballenger JC. Long-term pharmacologic treatment of panic disorder. J Clin Psychiatry. (1991) 52:18–23. 1995598

[121] Cohn JB Wilcox CS. Long-term comparison of alprazolam, lorazepam and placebo in patients with an anxiety disorder. Pharmacotherapy. (1984) 4:93–8. 10.1002/j.1875-9114.1984.tb03327.x 6144090

[122] Lucki I Rickels K. The behavioral effects of benzodiazepines following long-term use. Psychopharmacol Bull. (1986) 22:424–33. 2877474

[123] Lucki I Rickels K Geller AM. Chronic use of benzodiazepines and psychomotor and cognitive test performance. Psychopharmacology (Berl). (1986) 88:426–33. 10.1007/BF00178503 2871579

[124] Longone P Impagnatiello F Guidotti A Costa E. Reversible modification of GABAA receptor subunit mRNA expression during tolerance to diazepam-induced cognition dysfunction. Neuropharmacology. (1996) 35:1465–73. 10.1016/S0028-3908(96)00071-8 9014162

[125] Pomara N Lee SH Bruno D Silber T Greenblatt DJ Petkova E. Adverse performance effects of acute lorazepam administration in elderly long-term users: pharmacokinetic and clinical predictors. Prog Neuropsychopharmacol Biol Psychiatry. (2015) 56:129–35. 10.1016/j.pnpbp.2014.08.014 25195839 PMC4258460

[126] Paterniti S Dufouil C Alperovitch A. Long-term benzodiazepine use and cognitive decline in the elderly: the epidemiology of vascular aging study. J Clin Psychopharmacol. (2002) 22:285–93. 10.1097/00004714-200206000-00009 12006899

[127] McAndrews MP Kayumov L Phillipson R Shapiro CM. Self-report of memory and affective dysfunction in association with medication use in a sample of individuals with chronic sleep disturbance. Hum Psychopharmacol. (2000) 15:583–7. 10.1002/hup.226 12404610

[128] van Rijnsoever C Tauber M Choulli MK Keist R Rudolph U Mohler H. Requirement of 5-GABAA receptors for the development of tolerance to the sedative action of diazepam in mice. The J Neuroscience. (2004) 24:6785–90. 10.1523/JNEUROSCI.1067-04.2004 15282283 PMC6729721

[129] Duke AN Tiruveedhula VVNP Sharmin D Knutson DE Cook JM Platt DM. Tolerance and dependence following chronic alprazolam treatment in rhesus monkeys: Role of GABA. Drug Alcohol Depend. (2021) 228:108985. 10.1016/j.drugalcdep.2021.108985 34500240 PMC8595788

[130] Auta J Gatta E Davis JM Pandey SC Guidotti A. Potential role for histone deacetylation in chronic diazepam-induced downregulation of alpha1-GABAA receptor subunit expression. Pharmacol Res Perspect. (2018) 6:e00416. 10.1002/prp2.416 29951207 PMC6019704

[131] Perrault G Morel E Sanger DJ Zivkovic B. Lack of tolerance and physical dependence upon repeated treatment with the novel hypnotic zolpidem. J Pharmacol Exp Ther. (1992) 263:298–303. 1403792

[132] Elliot EE White JM. Precipitated and spontaneous withdrawal following administration of lorazepam but not zolpidem. Pharmacol Biochem Behav. (2000) 66:361–9. 10.1016/S0091-3057(00)00176-3 10880691

[133] Sanger DJ Zivkovic B. The discriminative stimulus properties of zolpidem, a novel imidazopyridine hypnotic. Psychopharmacology (Berl). (1986) 89:317–22. 10.1007/BF00174367 2873608

[134] Sanger DJ. Response decrement patterns after neuroleptic and non-neuroleptic drugs. Psychopharmacology (Berl). (1986) 89:98–104. 10.1007/BF00175198 2874584

[135] Stoops WW Rush CR. Differential effects in humans after repeated administrations of zolpidem and triazolam. Am J Drug Alcohol Abuse. (2003) 29:281–99. 10.1081/ADA-120020513 12765207

[136] Ebert B Anderson NJ Cremers TI Rasmussen S Vogel V Fahey JM. Gaboxadol – a different hypnotic profile with no tolerance to sleep EEG and sedative effects after repeated daily dosing. Pharmacol Biochem Behav. (2008) 90:113–22. 10.1016/j.pbb.2008.01.021 18304623

[137] Hoehns JD Perry PJ. Zolpidem: a nonbenzodiazepine hypnotic for treatment of insomnia. Clin Pharm. (1993) 12:814–28. 8275648

[138] Schlich D L'Heritier C Coquelin JP Attali P Kryrein HJ. Long-term treatment of insomnia with zolpidem: a multicentre general practitioner study of 107 patients. J Int Med Res. (1991) 19:271–9. 10.1177/030006059101900313 1670039

[139] Atack JR Wafford KA Street LJ Dawson GR Tye S Van Laere K. MRK-409 (MK-0343), a GABAA receptor subtype-selective partial agonist, is a non-sedating anxiolytic in preclinical species but causes sedation in humans. J Psychopharmacol. (2011) 25:314–28. 10.1177/0269881109354927 20147571

[140] Soumerai SB Simoni-Wastila L Singer C Mah C Gao X Salzman C. Lack of relationship between long-term use of benzodiazepines and escalation to high dosages. Psychiatr Serv. (2003) 54:1006–11. 10.1176/appi.ps.54.7.1006 12851438

[141] Silberman E Balon R Starcevic V Shader R Cosci F Fava GA. Benzodiazepines: it's time to return to the evidence. Br J Psychiatry. (2021) 218:125–7. 10.1192/bjp.2020.164 33040746

[142] Votava M Krsiak M Podhorna J Miczek KA. Alprazolam withdrawal and tolerance measured in the social conflict test in mice. Psychopharmacology (Berl). (2001) 157:123–30. 10.1007/s002130100784 11594436

[143] Follesa P Cagetti E Mancuso L Biggio F Manca A Maciocco E. Increase in expression of the GABA receptor a subunit gene A 4 induced by withdrawal of, but not by long-term treatment with, benzodiazepine full or partial agonists. Molec Brain Res. (2001) 92:138–48. 10.1016/S0169-328X(01)00164-4 11483250

[144] Lann MA Molina DK A. fatal case of benzodiazepine withdrawal. Am J Forensic Med Pathol. (2009) 30:177–9. 10.1097/PAF.0b013e3181875aa0 19465812

[145] Soyka M. Treatment of benzodiazepine dependence. N Engl J Med. (2017) 376:1147–57. 10.1056/NEJMra1611832 28328330

[146] Fluyau D Revadigar N Manobianco BE. Challenges of the pharmacological management of benzodiazepine withdrawal, dependence, and discontinuation. Therap Adv Psychopharmacol. (2018) 8:147–68. 10.1177/2045125317753340 29713452 PMC5896864

[147] Koob GF Volkow ND. Neurobiology of addiction: a neurocircuitry analysis. Lancet Psychiatry. (2016) 3:760–73. 10.1016/S2215-0366(16)00104-8 27475769 PMC6135092

[148] Uusi-Oukari M Korpi ER. Regulation of GABAA receptor subunit expression by pharmacological agents. Pharmacol Rev. (2010) 62:97–135. 10.1124/pr.109.002063 20123953

[149] Holt RA Bateson AN Martin IL. Chronic treatment with diazepam or abecarnil differentially affects the expression of GABAA receptor subunit mRNAs in the rat cortex. Neuropharmacology. (1996) 35:1457–63. 10.1016/S0028-3908(96)00064-0 9014161

[150] Holt RA Martin IL Bateson AN. Chronic diazepam exposure decreases transcription of the rat GABA(A) receptor gamma2-subunit gene. Brain Res Mol Brain Res. (1997) 48:164–6. 10.1016/S0169-328X(97)00129-0 9379839

[151] Holt RA Bateson AN Martin IL. Decreased GABA enhancement of benzodiazepine binding after a single dose of diazepam. J Neurochem. (1999) 72:2219–22. 10.1046/j.1471-4159.1999.0722219.x 10217306

[152] Zhao TJ Chiu TH Rosenberg HC. Reduced expression of gamma-aminobutyric acid type A/benzodiazepine receptor gamma 2 and alpha 5 subunit mRNAs in brain regions of flurazepam-treated rats. Mol Pharmacol. (1994) 45:657–63. 10.1007/BF02736732 8183244

[153] Tietz EI Chiu TH Rosenberg HC. Regional GABA/benzodiazepine receptor/chloride channel coupling after acute and chronic benzodiazepine treatment. Eur J Pharmacol. (1989) 167:57–65. 10.1016/0014-2999(89)90747-4 2476326

[154] Tietz EI Huang XG Weng XJ Rosenberg HC Chiu TH. Expression of alpha(1), alpha(5), and gamma(2) GABA(A) receptor subunit messenger RNAs as measured in situ in rat hippocampus and cortex following chronic flurazepam administration. J Molec Neurosci. (1993) 4:277–92. 10.1007/BF02821559 7917836

[155] Follesa P Mancuso L Biggio F Cagetti E Franco M Trapani G. Changes in GABA(A) receptor gene expression induced by withdrawal of, but not by long-term exposure to, zaleplon or zolpidem. Neuropharmacology. (2002) 42:191–8. 10.1016/S0028-3908(01)00167-8 11804615

[156] Allison C Pratt JA. Neuroadaptive processes in GABAergic and glutamatergic systems in benzodiazepine dependence. Pharmacol Ther. (2003) 98:171–95. 10.1016/S0163-7258(03)00029-9 12725868

[157] Das P Lilly SM Zerda R Gunning WT Alvarez FJ Tietz EI. Increased AMPA receptor glur1subunit incorporation in rat hippocampal CA1 synapses during benzodiazepine withdrawal. J Comp Neurol. (2008) 511:832–46. 10.1002/cne.21866 18924138 PMC2593095

[158] Das P Zerda R Alvarez FJ Tietz EI. Immunogold electron microscopic evidence of differential regulation of GluN1, GluN2A, and GluN2B, NMDA-type glutamate receptor subunits in rat hippocampal CA1 synapses during benzodiazepine withdrawal. J Comp Neurol. (2010) 518:4311–28. 10.1002/cne.22458 20853509 PMC2943829

[159] Shen G Tietz EI. Down-regulation of synaptic GluN2B Subunit-Containing N-methyl-D-aspartate receptors: a physiological brake on CA1 Neuron -Amino-3-hydroxy-5-methyl-4-isoxazolepropionic acid hyperexcitability during benzodiazepine withdrawal. J Pharmacol Exper Therap. (2011) 336:265–73. 10.1124/jpet.110.174235 20935233 PMC3014299

[160] Shen G Van Sickle BJ Tietz E. Calcium/calmodulin-dependent protein kinase ii mediates hippocampal glutamatergic plasticity during benzodiazepine withdrawal. Neuropsychopharmacology. (2010) 35:1897–909. 10.1038/npp.2010.61 20445501 PMC2904841

[161] Song J Shen GF Greenfield LJ Tietz EI. Benzodiazepine withdrawal-induced glutamatergic plasticity involves up-regulation of GluR1-containing alpha-amino-3-hydroxy-5-methylisoxazole-4-propionic acid receptors in hippocampal CA1 neurons. J Pharmacol Exper Therap. (2007) 322:569–81. 10.1124/jpet.107.121798 17510319

[162] Vekovischeva OY Neuvonen PJ Korpi ER. Reduced benzodiazepine tolerance, but increased flumazenil-precipitated withdrawal in AMPA-receptor GluR-A subunit-deficient mice. Pharmacol. Biochem Behav. (2009) 92:283–90. 10.1016/j.pbb.2008.12.015 19141300

[163] Okamoto Y Itoh Y Murata Y Kobayashi D Hosoi M Mine K. Reduction of group II metabotropic glutamate receptors during development of benzodiazepine dependence. Pharmacology. (2013) 91:145–52. 10.1159/000346440 23392308

[164] Steppuhn KG Turski L. Diazepam dependence prevented by glutamate antagonists. Proc Natl Acad Sci U S A. (1993) 90:6889–93. 10.1073/pnas.90.14.6889 8341715 PMC47038

[165] Van Sickle BJ Cox AS Schak K Greenfield LJ Tietz EI. Chronic benzodiazepine administration alters hippocampal CA1 neuron excitability: NMDA receptor function and expression(1). Neuropharmacology. (2002) 43:595–606. 10.1016/S0028-3908(02)00152-1 12367605

[166] Van Sickle BJ Tietz EI. Selective enhancement of AMPA receptor-mediated function in hippocampal CA1 neurons from chronic benzodiazepine-treated rats. Neuropharmacology. (2002) 43:11–27. 10.1016/S0028-3908(02)00065-5 12213255

[167] Van Sickle BJ Xiang K Tietz EI. Transient plasticity of hippocampal CA1 neuron glutamate receptors contributes to benzodiazepine withdrawal-anxiety. Neuropsychopharmacology. (2004) 29:1994–2006. 10.1038/sj.npp.1300531 15266351

[168] Talarek S Listos J Fidecka S. Effect of nitric oxide synthase inhibitors on benzodiazepine withdrawal in mice and rats. Pharmacol Rep. (2011) 63:680–9. 10.1016/S1734-1140(11)70579-5 21857078

[169] Listos J Malec D Fidecka S. Influence of adenosine receptor agonists on benzodiazepine withdrawal signs in mice. Eur J Pharmacol. (2005) 523:71–8. 10.1016/j.ejphar.2005.07.025 16226742

[170] Singh L Field MJ Vass CA Hughes J Woodruff GN. The antagonism of benzodiazepine withdrawal effects by the selective cholecystokininB receptor antagonist CI-988. Br J Pharmacol. (1992) 105:8–10. 10.1111/j.1476-5381.1992.tb14201.x 1350747 PMC1908629

[171] Stephens DN King SL Lambert JJ Belelli D Duka T. GABA(A) receptor subtype involvement in addictive behaviour. Genes Brain Behav. (2017) 16:149–84. 10.1111/gbb.12321 27539865

[172] Wallner M Hanchar HJ Olsen RW. Low dose acute alcohol effects on GABA A receptor subtypes. Pharmacol Ther. (2006) 112:513–28. 10.1016/j.pharmthera.2006.05.004 16814864 PMC2847605

[173] Olsen RW Hanchar HJ Meera P Wallner M GABAA. receptor subtypes: the “one glass of wine” receptors. Alcohol. (2007) 41:201–9. 10.1016/j.alcohol.2007.04.006 17591543 PMC2852584

[174] Liang J Zhang N Cagetti E Houser CR Olsen RW Spigelman I. Chronic intermittent ethanol-induced switch of ethanol actions from extrasynaptic to synaptic hippocampal GABAA receptors. J Neurosci. (2006) 26:1749–58. 10.1523/JNEUROSCI.4702-05.2006 16467523 PMC6793625

[175] Zeng K Xie A Zhang X Zhong B Liu X Hao W. Chronic alcohol treatment-induced GABA-Aalpha5 Histone H3K4 trimethylation upregulation leads to increased GABA-Aalpha5 expression and susceptibility to alcohol addiction in the offspring of wistar rats. Front Psychiatry. (2018) 9:468. 10.3389/fpsyt.2018.00468 30405449 PMC6208097

[176] Gatta E Auta J Gavin DP Bhaumik DK Grayson DR Pandey SC. Emerging role of one-carbon metabolism and DNA methylation enrichment on delta-containing GABAA receptor expression in the cerebellum of subjects with alcohol use disorders (AUD). Int J Neuropsychopharmacol. (2017) 20:1013–26. 10.1093/ijnp/pyx075 29020412 PMC5716183

[177] Olsen RW Liang J Cagetti E Spigelman I. Plasticity of GABAA receptors in brains of rats treated with chronic intermittent ethanol. Neurochem Res. (2005) 30:1579–88. 10.1007/s11064-005-8836-6 16362777

[178] Werner DF Porcu P Boyd KN O'Buckley TK Carter JM Kumar S. Ethanol-induced GABAA receptor alpha4 subunit plasticity involves phosphorylation and neuroactive steroids. Mol Cell Neurosci. (2016) 72:1–8. 10.1016/j.mcn.2016.01.002 26805653 PMC4932829

[179] Edenberg HJ Dick DM Xuei X Tian H Almasy L Bauer LO. Variations in GABRA2, encoding the alpha 2 subunit of the GABA(A) receptor, are associated with alcohol dependence and with brain oscillations. Am J Hum Genet. (2004) 74:705–14. 10.1086/383283 15024690 PMC1181946

[180] Covault J Gelernter J Hesselbrock V Nellissery M Kranzler HR. Allelic and haplotypic association of GABRA2 with alcohol dependence. Am J Med Genet Part B, Neuropsych Genet. (2004) 129B:104–9. 10.1002/ajmg.b.30091 15274050

[181] Fehr C Sander T Tadic A Lenzen KP Anghelescu I Klawe C. Confirmation of association of the GABRA2 gene with alcohol dependence by subtype-specific analysis. Psychiatr Genet. (2006) 16:9–17. 10.1097/01.ypg.0000185027.89816.d9 16395124

[182] Soyka M Preuss UW Hesselbrock V Zill P Koller G Bondy B. GABA-A2 receptor subunit gene (GABRA2) polymorphisms and risk for alcohol dependence. J Psychiatr Res. (2008) 42:184–91. 10.1016/j.jpsychires.2006.11.006 17207817

[183] Enoch MA. The role of GABA(A) receptors in the development of alcoholism. Pharmacol Biochem Behav. (2008) 90:95–104. 10.1016/j.pbb.2008.03.007 18440057 PMC2577853

[184] Borghese CM Harris RA. Alcohol dependence and genes encoding alpha2 and gamma1 GABAA receptor subunits: insights from humans and mice. Alcohol Res. (2012) 34:345–53. 23134051 10.35946/arcr.v34.3.10 PMC3860398

[185] Bierut LJ Agrawal A Bucholz KK Doheny KF Laurie C Pugh E. A genome-wide association study of alcohol dependence. Proc Natl Acad Sci U S A. (2010) 107:5082–7. 10.1073/pnas.0911109107 20202923 PMC2841942

[186] Treutlein J Cichon S Ridinger M Wodarz N Soyka M Zill P. Genome-wide association study of alcohol dependence. Arch Gen Psychiatry. (2009) 66:773–84. 10.1001/archgenpsychiatry.2009.83 19581569 PMC4229246

[187] Enoch MA Zhou Z Kimura M Mash DC Yuan Q Goldman D. GABAergic gene expression in postmortem hippocampus from alcoholics and cocaine addicts; corresponding findings in alcohol-naive P and NP rats. PLoS ONE. (2012) 7:e29369. 10.1371/journal.pone.0029369 22253714 PMC3258238

[188] Loh EW Smith I Murray R McLaughlin M McNulty S Ball D. Association between variants at the GABAAbeta2, GABAAalpha6 and GABAAgamma2 gene cluster and alcohol dependence in a Scottish population. Mol Psychiatry. (1999) 4:539–44. 10.1038/sj.mp.4000554 10578235

[189] Park C-S Park S-Y Lee C-S Sohn J-W Hahn G-H Kim B-J. Association between alcoholism and the genetic polymorphisms of the GABAA receptor genes on chromosome 5q33-34 in Korean population. J Korean Med Sci. (2006) 21:533–8. 10.3346/jkms.2006.21.3.533 16778401 PMC2729963

[190] Enoch MA Hodgkinson CA Yuan Q Shen PH Goldman D Roy A. The influence of GABRA2, childhood trauma, and their interaction on alcohol, heroin, and cocaine dependence. Biol Psychiatry. (2010) 67:20–7. 10.1016/j.biopsych.2009.08.019 19833324 PMC2964936

[191] Dixon CI Morris HV Breen G Desrivieres S Jugurnauth S Steiner RC. Cocaine effects on mouse incentive-learning and human addiction are linked to alpha2 subunit-containing GABAA receptors. Proc Natl Acad Sci U S A. (2010) 107:2289–94. 10.1073/pnas.0910117107 20133874 PMC2836671

[192] Smelson D Yu L Buyske S Gonzalez G Tischfield J Deutsch CK. Genetic association of GABA-A receptor alpha-2 and mu opioid receptor with cocaine cue-reactivity: evidence for inhibitory synaptic neurotransmission involvement in cocaine dependence. American Journal on Addictions. (2012) 21:411–5. 10.1111/j.1521-0391.2012.00253.x 22882391 PMC3425941

[193] Dixon CI Walker SE Swinny J Belelli D Lambert JJ King SL. Early-life stress influences acute and sensitized responses of adult mice to cocaine by interacting with GABAA alpha2 receptor expression. Behav Pharmacol. (2019) 30:272–81. 10.1097/FBP.0000000000000466 30724801

[194] Mitchell SJ Maguire EP Cunningham L Gunn BG Linke M Zechner U. Early-life adversity selectively impairs alpha2-GABA(A) receptor expression in the mouse nucleus accumbens and influences the behavioral effects of cocaine. Neuropharmacology. (2018) 141:98–112. 10.1016/j.neuropharm.2018.08.021 30138693 PMC6178871

[195] Lilly SM Tietz EI. Chronic cocaine differentially affects diazepam's anxiolytic and anticonvulsant actions. Relationship to GABA(A) receptor subunit expression. Brain Res. (2000) 882:139–48. 10.1016/S0006-8993(00)02858-4 11056193

[196] Huggett SB Stallings MC. Genetic architecture and molecular neuropathology of human cocaine addiction. J Neurosci. (2020) 40:5300–13. 10.1523/JNEUROSCI.2879-19.2020 32457073 PMC7329314

